# Impact of Undetected Comorbidity on Treatment and Outcomes of Breast Cancer

**DOI:** 10.1155/2014/970780

**Published:** 2014-02-13

**Authors:** Robert I. Griffiths, Michelle L. Gleeson, José M. Valderas, Mark D. Danese

**Affiliations:** ^1^Nuffield Department of Primary Care Health Sciences, University of Oxford, 23-38 Hythe Bridge Street, 2nd Floor, Oxford OX1 2ET, UK; ^2^Division of General Internal Medicine, Johns Hopkins University School of Medicine, Baltimore, MD 21205, USA; ^3^Outcomes Insights, 340 North Westlake Blvd, Suite 200, Westlake Village, CA 91362, USA

## Abstract

Preexisting comorbidity adversely impacts breast cancer treatment and outcomes. We examined the incremental impact of comorbidity undetected until cancer. We followed breast cancer patients in SEER-Medicare from 12 months before to 84 months after diagnosis. Two comorbidity indices were constructed: the National Cancer Institute index, using 12 months of claims before cancer, and a second index for previously undetected conditions, using three months after cancer. Conditions present in the first were excluded from the second. Overall, 6,184 (10.1%) had ≥1 undetected comorbidity. Chronic obstructive pulmonary disease (38%) was the most common undetected condition. In multivariable analyses that adjusted for comorbidity detected before cancer, older age, later stage, higher grade, and poor performance status all were associated with higher odds of ≥1 undetected comorbidity. In stage I–III cancer, undetected comorbidity was associated with lower adjusted odds of receiving adjuvant chemotherapy (Odds Ratio (OR) = 0.81, 95% Confidence Interval (CI) 0.73–0.90, *P* < 0.0001; OR = 0.38, 95% CI 0.30–0.49, *P* < 0.0001; index score 1 or ≥2, respectively), and with increased mortality (Hazard Ratio (HR) = 1.45, 95% CI 1.38–1.53, *P* < 0.0001; HR = 2.38, 95% CI 2.18–2.60, *P* < 0.0001; index score 1 or ≥2). Undetected comorbidity is associated with less aggressive treatment and higher mortality in breast cancer.

## 1. Introduction

Comorbidity adversely impacts the treatment [1–7] and outcomes [1, 3, 6, 8–22] of breast cancer, especially in older patients. For instance, studies have shown that breast cancer patients with previously identified comorbidity are less likely to receive adjuvant chemotherapy [2, 4, 5] and have higher mortality [8]. Many such studies from the United States are based on the Surveillance, Epidemiology, and End Results cancer registry linked to health insurance claims (SEER-Medicare) [1, 2, 5, 6, 17–20], where a common approach is first to identify conditions appearing in claims prior to cancer and then to use these conditions to construct a National Cancer Institute (NCI) Comorbidity Index [23], which is an adaptation of the Charlson index (CCI) [24].

Most studies based on SEER-Medicare include some measures of comorbidity, and in a random sample of all SEER-Medicare studies published between 2006 and 2011, 23/30 (77%) included the NCI Comorbidity Index. One important limitation of this index, however, is that it ignores conditions that are undetected in claims until after cancer is diagnosed. Recently, we examined the incidence of undetected comorbidity in breast cancer [25] and found that many chronic conditions included in the NCI Comorbidity Index, such as chronic obstructive pulmonary disease (COPD), congestive heart failure (CHF), diabetes, and cerebrovascular disease (CVD), remained undetected in claims until 1–3 months after cancer.

Undetected comorbidity could have additional implications for breast cancer treatment and outcomes. Therefore, in this study we sought to describe risk factors for comorbidity that remained undetected until breast cancer, and to assess the added impact of undetected comorbidity on breast cancer treatment and outcomes.

## 2. Methods

### 2.1. Data

Presently SEER-Medicare [26] includes all Medicare-eligible persons from 16 of the 17 SEER registries through 2005 and their Medicare claims for Part A (inpatient) and Part B (outpatient and physician services) through 2007.

### 2.2. Patients

Patients were included if they were diagnosed with breast cancer between 2001-01-01 and 2005-12-31, had only one primary cancer diagnosed, and had at least 12 months of Medicare Part A and Part B coverage prior to cancer. They were excluded for the following reasons: male breast cancer, cancer diagnosed before age 65, diagnosis made by death certificate or autopsy, death within the first month following diagnosis, or Medicare enrollment less than 12 months before diagnosis. Patients were followed up from 12 months before cancer until the end of the claims period (2007-12-31) or death, whichever came first. Since SEER reports only the month of cancer diagnosis, the first day of that month was assigned as the date of diagnosis.

#### 2.2.1. Variables

Patients were described according to their demographic, clinical, and socioeconomic characteristics [26, 27]. SEER does not include measures of performance status, such as Eastern Cooperative Oncology Group. Instead, we used Medicare claims to identify several indictors of poor performance status, including the use of oxygen and related respiratory therapy supplies, wheelchair and supplies, home health agency services, and skilled nursing facility admissions, all from 12 months before until 30 days after breast cancer diagnosis [28].

The SEER-Medicare dataset contains information from the 2000 US Census, reported at the tract level in which the patient lives, for the percent of the population living in poverty and the percent of those aged ≥25 years with some college. We used these as indicators of the socioeconomic status of individual patients in the cohort.

#### 2.2.2. Comorbidity

We constructed two indices of comorbidity. In SEER-Medicare, the gold standard for documenting the presence of comorbidity prior to cancer is the NCI Comorbidity Index [23], which is usually constructed based on claims from 12 months to one month before cancer diagnosis. Diagnosis and procedure codes are used to identify the 15 noncancer comorbidities in the CCI [24]. A weight is then assigned to each condition, and the weights are summed to obtain the index score for each patient. We followed this approach to capture comorbidity detected prior to cancer and classified patients as having a score =0, 1, or ≥2.

We applied the same algorithms used in the NCI Comorbidity Index to construct an index of undetected comorbidity, which was based on claims from the month of cancer diagnosis up to two months after. For each patient, conditions identified prior to cancer were excluded from the undetected comorbidity index. If chemotherapy or radiation began within three months following cancer diagnosis, the observation period for the undetected comorbidity index was truncated at that time to minimize the risk of misclassifying treatment-related adverse events as undetected conditions. As above, patients were classified as having a score = 0, 1, or ≥2. Since the minimum weight assigned to any condition in either index is one (1), for clarity the term “≥ 1 condition/comorbidity” is used in the Results to describe patients with a score on either the previously detected or undetected comorbidity index of ≥1.

#### 2.2.3. Adjuvant Chemotherapy

Adjuvant chemotherapy was identified based on Healthcare Common Procedure Coding (HCPCS) “J” codes as recommended by the NCI [29]. The first claim for chemotherapy had to appear within 180 days of cancer diagnosis for the patient to be classified as having received chemotherapy.

### 2.3. Mortality

The date of death was assigned by using the Medicare date if available. When the Medicare date was missing but the SEER date was available, the SEER date was used. All other patients were assumed to be alive at the end of the observation period. The cause of death was classified as cancer or other cause.

### 2.4. Statistical Analysis

Multivariable analyses were performed to examine risk factors for undetected comorbidity, as well as associations between undetected comorbidity, adjuvant chemotherapy, and mortality. Both indices of comorbidity were included in analyses of treatment and mortality to assess the incremental impact of undetected comorbidity. The multivariable analysis of adjuvant chemotherapy was limited to those with stage I–III disease [5]. All multivariable survival analyses included only those who survived at least 3 months after cancer diagnosis. Final models were selected through a process of forward, stepwise regression, taking into account statistical significance, interactions among covariables, and whether a nonstatistically significant finding was informative. In almost all instances, at least one level of each variable included in the final model was statistically significantly different from the reference category of that variable. In all instances, prior comorbidity and previously undetected comorbidity were included in the final multivariable model. However, we compared the magnitude and significance of the coefficients on the previously undetected comorbidity in models that included versus excluded prior comorbidity as a covariable to assess the level of colinearity between these two.

## 3. Results

There were 61,002 patients in the final cohort, of whom 6,184 (10%) had ≥1 undetected comorbidity. The mean (standard deviation) age was 76.0 (6.8), 54.6% were aged >75 years, 61% were diagnosed with *in situ* or stage I disease, 14% had ≥1 indicator of poor performance, and 33% had ≥1 comorbidity detected prior to cancer (Table 1). Those with ≥1 undetected comorbidity also were more likely to have ≥1 condition detected prior to cancer.

### 3.1. Undetected Comorbidity

Among the 6,184 patients with ≥1 undetected comorbidity, there were a total of 7,593 conditions. The most common were COPD (33% [2,023/6,184] of patients), CHF (18%), diabetes (18% without complications; 4% with complications), myocardial infarction (14%), and CVD (10%). In multivariable logistic regression, factors associated with having ≥1 undetected condition were older age, ≥1 comorbidity detected prior to cancer, ≥1 indicator of poor performance status, advanced stage, histological grade, living in a census tract with more poverty, and living in an urban area. Other race/ethnicity (compared to white) and later year of cancer diagnosis were associated with lower odds of having ≥1 undetected condition (Table 2).

### 3.2. Chemotherapy

In multivariable analysis, undetected comorbidity was associated with lower adjusted odds of receiving adjuvant chemotherapy (Odds Ratio (OR) = 0.81, 95% Confidence Interval (CI) 0.73–0.90, *P* < 0.0001; OR = 0.38, 95% CI 0.30–0.49, *P* < 0.0001, for undetected comorbidity index score 1 or ≥2, resp.) (Table 3). Other factors associated with lower odds were older age, later year of diagnosis, having ≥2 conditions detected prior to cancer, being either estrogen and/or progesterone receptor positive, and living in a census tract with >12% poverty. Factors associated with higher odds included advanced stage and higher grade.

### 3.3. Survival

**Figure 1:**
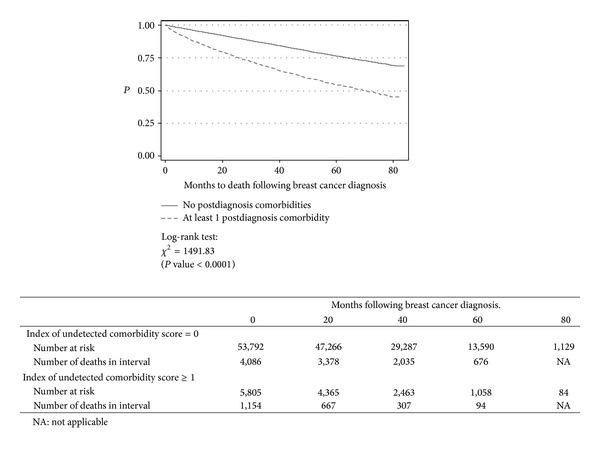
Unadjusted Survival.

Overall, there were 13,208 (22% of cohort) deaths: 5,191 (39% of all deaths) due to cancer; 5,823 (44%) due to other causes; and (17%) unspecified. Among those with ≥1 undetected comorbidity, 42% (2,575) died during the observation period compared to 19% (10,633) of those with no undetected conditions, and the unadjusted mortality rate was significantly higher (*P* < 0.0001) (Figure 1).

In multivariable survival analysis, having undetected comorbidity was associated with increased all-cause mortality (Hazard Ratio (HR) = 1.45, 95% CI 1.38–1.53, *P* < 0.0001; HR = 2.38, 95% CI 2.18–2.60, *P* < 0.0001, for undetected comorbidity index score 1 or ≥2, resp.), cancer mortality (HR = 1.32 and HR = 1.67 for score 1 or ≥2), and other-cause mortality (HR = 1.58 and HR = 3.31 for score 1 or ≥2) (Table 4). In most instances, coefficients for undetected comorbidity were comparable to or larger than those for comorbidity detected prior to cancer.

### 3.4. Models with versus without Previously Detected Comorbidity

There was little difference in the magnitude and significance of the coefficients for the previously undetected comorbidity for multivariable models that included versus excluded prior comorbidity as a covariable. For instance, the coefficients for the chemotherapy model were 0.80 and 0.37 for undetected comorbidity scores of 1 and ≥2, respectively, in the model that *excluded* prior comorbidity compared to 0.81 and 0.38 in the model that *included* prior comorbidity (Table 3).

## 4. Discussion

We conducted an observational cohort study using SEER-Medicare to describe risk factors for comorbidity that remained undetected until breast cancer and to assess the added impact of undetected comorbidity on breast cancer treatment and outcomes. Advanced age, later stage diagnosis, poor performance status, presence of comorbidity identified prior to cancer, and poverty all were associated with increased risk of undetected comorbidity. The strong monotonic association with cancer stage, as well as the association with poverty, suggests the existence of shared risk factors, such as poor health system contact prior to cancer diagnosis, [30, 31] resulting in delayed detection of both cancer and other underlying chronic conditions including COPD, CHF, and diabetes. One important implication of our study is that those with other established risk factors for poor outcomes in breast cancer, including advanced age, later stage, and poor performance status, also are at greatest risk for undetected comorbidity.

Patients with undetected comorbidity were less likely to receive adjuvant chemotherapy. Furthermore, the effect sizes were larger for undetected comorbidity than for comorbidity detected prior to cancer. For example the Odds Ratio for an undetected comorbidity index score ≥2 was 0.38 compared to 0.74 for a score ≥2 on the index of conditions prior to cancer. Since identification of conditions was based on claims for medical services, one hypothesis is that undetected conditions were more severe because they had not been treated. Others have speculated that one way comorbidity can influence treatment and outcomes in cancer is by distracting the healthcare team from the appropriate management of both [32] cancer and other conditions. The fact that effect sizes were larger for undetected conditions suggest that these may pose greater challenges for the healthcare team than those that have a history of being treated prior to cancer.

Undetected comorbidity also was associated with increased overall cancer and other-cause mortality. The impact on other-cause mortality was larger than on cancer mortality. However, all six HRs in the three survival analyses were highly statistically significant, and, in general, HRs for undetected comorbidity were larger than those for comorbidity detected prior to cancer. Again, this supports the hypothesis that undetected comorbidity may be more severe and that it may pose greater challenges to the healthcare team than conditions appearing in claims and, by implication, treated prior to cancer.

It is likely that the impact of undetected comorbidity on cancer mortality is partially explained by its direct impact on the likelihood of receiving adjuvant chemotherapy. The link between adjuvant chemotherapy and survival in breast cancer is well established, and adjuvant chemotherapy was not included in our survival analyses to avoid over adjustment of the models.

Our study has several limitations. First, as discussed above, we cannot rule out the possibility that unobserved factors may have confounded observed associations between the presence of undetected comorbidity and several of the observed risk factors, notably cancer stage at diagnosis. Second, both comorbidity indices in our study are based on claims and consequently have less-than-perfect sensitivity and specificity for the conditions of interest. Therefore, conditions that simply were not found in claims prior to cancer due to limitations of the algorithms in the NCI Comorbidity Index, but which actually had been detected and possibly treated at an earlier date, may have been misclassified as undetected. Third, since SEER includes only the month and not the day of cancer diagnosis, we elected to assign the first day of the month as the date of cancer diagnosis. Had we elected to assign the last day of the month, all conditions classified as previously undetected by virtue of first appearing in the month of cancer diagnosis would have been reclassified as previously detected.

## 5. Conclusions

Limitations notwithstanding, our findings indicate that those patients with established risk factors for poor outcomes in breast cancer, including advanced age, later stage, and poor performance status, also are at greatest risk for undetected comorbidity. Furthermore, undetected comorbidity appears to confer greater additional risk of poor outcomes than comorbidity detected prior to cancer, suggesting that it may be more severe and that it may pose additional challenges to the healthcare team. However, these hypotheses would require further investigation.

## Figures and Tables

**Table 1:** Patient characteristics.

			Index of undetected comorbidity				
	Overall		Score				
	(*n* = 61,002)		0		≥1		*P* value
	*n*	%	(*n* = 54,818)		(*n* = 6,184)		
			*n*	%	*n*	%	
Age at cancer diagnosis (years)							
66–70	12,361	20.3	11,412	20.8	949	15.4	<0.0001
71–75	15,368	25.2	14,036	25.6	1,332	21.5	
76–80	15,108	24.8	13,576	24.8	1,532	24.8	
>80	18,165	29.8	15,794	28.8	2,371	38.3	
Race/ethnicity							
White	52,270	85.7	47,048	85.8	5,222	84.4	<0.0001
Black	4,188	6.9	3,639	6.6	549	8.9	
Hispanic	2,249	3.7	2,016	3.7	233	3.8	
Other	2,295	3.8	2,115	3.9	180	2.9	
Year of cancer diagnosis							
2001	12,660	20.8	11,322	20.7	1,338	21.6	0.12
2002	12,452	20.4	11,166	20.4	1,286	20.8	
2003	12,086	19.8	10,845	19.8	1,241	20.1	
2004	11,929	19.6	10,765	19.6	1164	18.8	
2005	11,875	19.5	10,720	19.6	1155	18.7	
Index of prior comorbidity score (NCI Comorbidity Index)							
0	41,001	67.2	37,365	68.2	3,636	58.8	<0.0001
1	13,480	22.1	11,952	21.8	1,528	24.7	
≥2	6,521	10.7	5,501	10.0	1020	16.5	
Stage at diagnosis							
*In* *situ*	10,167	16.7	9,618	17.6	549	8.8	<0.0001
I	26,754	43.9	24,578	44.8	2,176	35.1	
II	17,165	28.1	15,080	27.5	2,085	33.7	
III	4,096	6.7	3,428	6.3	668	10.8	
IV	2,820	4.6	2,114	3.9	706	11.4	
Estrogen (ER) and progesterone (PR) receptor status							
ER and PR positive	31,419	51.5	28,347	51.7	3,072	49.7	<0.0001
ER or PR positive	8,081	13.3	7,203	13.1	878	14.2	
ER and PR negative	7,165	11.8	6,353	11.6	812	13.1	
Unknown/missing	14,337	23.5	12,915	23.6	1,422	23.0	
Histological grade							
1	12,419	20.4	11,403	20.8	1,016	16.4	<0.0001
2	23,407	38.4	21,090	38.5	2,317	37.5	
3	15,291	25.1	13,528	24.7	1,763	28.5	
4	1,881	3.1	1,710	3.1	171	2.8	
Missing/unknown	8,004	13.1	7,087	12.9	917	14.8	
Indicators of poor performance							
0	52,460	86.0	47,599	86.8	4,861	78.6	<0.0001
≥1	8,542	14.0	7,219	13.2	1,323	21.4	
Percent in census tract with some college							
<25%	21,117	34.6	18,822	34.4	2,295	37.1	<0.0001
≥25%	39,885	65.4	35,996	65.7	3,889	62.9	
Percent in census tract living in poverty							
<5%	19,951	32.7	18,276	33.3	1,675	27.1	<0.0001
5–7%	8,718	14.3	7,919	14.5	799	12.9	
8–12%	13,566	22.2	12,177	22.2	1,389	22.5	
>12%	18,767	30.8	16,446	30.0	2,321	37.5	
Type of geographic area							
Large metropolitan	34,481	56.5	31,090	56.7	3,391	54.8	<0.0001
Metropolitan	17,568	28.8	15,840	28.9	1,728	27.9	
Urban	3,674	6.0	3,242	5.9	432	7.0	
Less urban/rural	5,279	8.7	4,646	8.5	633	10.2	

**Table 2:** Risk of undetected comorbidity.

	OR	95% CI		P value
		Lower	Upper	
Age at cancer diagnosis (years)				
66–70	Reference category			
71–75	1.12	1.02	1.22	0.02
76–80	1.28	1.17	1.39	<0.0001
>80	1.53	1.41	1.66	<0.0001
Race/ethnicity				
White	Reference category			
Black	1.04	0.94	1.16	0.41
Hispanic	0.94	0.81	1.08	0.39
Other	0.83	0.71	0.97	0.02
Year of cancer diagnosis				
2001	Reference category			
2002	0.99	0.91	1.07	0.75
2003	0.98	0.90	1.07	0.68
2004	0.93	0.85	1.01	0.08
2005	0.92	0.84	1.00	0.05
Index of prior comorbidity score (NCI Comorbidity Index)				
0	Reference category			
1	1.20	1.13	1.28	<0.0001
≥2	1.50	1.38	1.63	<0.0001
Indicators of poor performance				
0	Reference category			
≥1	1.30	1.21	1.40	<0.0001
Stage at diagnosis				
*In* *situ*	Reference category			
I	1.66	1.49	1.85	<0.0001
II	2.40	2.15	2.67	<0.0001
III	3.26	2.87	3.71	<0.0001
IV	5.51	4.85	6.26	<0.0001
Histological grade				
Well differentiated	Reference category			
Moderately differentiated	1.11	1.02	1.20	0.01
Poorly differentiated	1.15	1.05	1.26	<0.01
Undifferentiated/anaplastic	1.25	1.05	1.50	0.01
Missing/unknown	1.14	1.03	1.27	0.01
Estrogen (ER) and progesterone (PR) receptor status				
ER and PR negative	Reference category			
ER or PR positive	1.05	0.94	1.16	0.39
ER and PR positive	1.00	0.92	1.09	1.00
Unknown/missing	1.14	1.03	1.27	0.01
Percent in census tract with some college				
<25%	Reference category			
≥25%	0.99	0.93	1.05	0.70
Percent in census tract living in poverty				
<5%	Reference category			
5–7%	1.07	0.98	1.17	0.12
8–12%	1.17	1.08	1.27	<0.0001
>12%	1.34	1.25	1.45	<0.0001
Type of geographic area				
Large metropolitan	Reference category			
Metropolitan	0.97	0.91	1.04	0.42
Urban	1.14	1.02	1.28	0.02
Less urban/rural	1.09	0.99	1.20	0.08

OR: Odds Ratio; CI: Confidence Interval.

**Table 3:** Adjuvant chemotherapy.

	OR	95% CI		*P* value
		Lower	Upper	
Age at cancer diagnosis (years)				
66–70	Reference category			
71–75	0.55	0.51	0.59	<0.0001
76–80	0.25	0.23	0.27	<0.0001
>80	0.05	0.04	0.05	<0.0001
Race/ethnicity				
White	Reference category			
Black	0.94	0.84	1.05	0.27
Hispanic	1.15	0.99	1.32	0.06
Other	1.13	0.98	1.31	0.10
Year of cancer diagnosis				
2001	Reference category			
2002	0.95	0.87	1.04	0.26
2003	0.86	0.79	0.94	<0.001
2004	0.88	0.80	0.96	<0.01
2005	0.79	0.72	0.86	<0.0001
Index of prior comorbidity score (NCI Comorbidity Index)				
0	Reference category			
1	0.96	0.90	1.03	0.28
≥2	0.74	0.66	0.82	<0.0001
Index of undetected comorbidity score				
0	Reference category			
1	0.81	0.73	0.90	<0.0001
≥2	0.38	0.30	0.49	<0.0001
Indicators of poor performance				
0	Reference category			
≥1	0.56	0.51	0.62	<0.0001
Stage at diagnosis				
I	Reference category			
II	8.73	8.17	9.34	<0.0001
III	21.87	19.83	24.12	<0.0001
Histological grade				
Well differentiated	Reference category			
Moderately differentiated	1.58	1.44	1.72	<0.0001
Poorly differentiated	2.54	2.31	2.79	<0.0001
Undifferentiated/anaplastic	2.01	1.60	2.53	<0.0001
Missing/unknown	1.51	1.33	1.71	<0.0001
Estrogen (ER) and progesterone (PR) receptor status				
ER and PR negative	Reference category			
Either ER or PR positive	0.32	0.29	0.35	<0.0001
Both ER and PR positive	0.24	0.22	0.26	<0.0001
Unknown/missing	0.30	0.27	0.34	<0.0001
Percent in census tract with some college				
<25%	Reference category			
≥25%	1.02	0.95	1.08	0.65
Percent in census tract living in poverty				
<5%	Reference category			
5–7%	0.93	0.85	1.02	0.13
8–12%	0.93	0.86	1.01	0.08
>12%	0.91	0.84	0.98	0.01
Type of geographic area				
Large metropolitan	Reference category			
Metropolitan	1.12	1.05	1.20	<0.01
Urban	1.14	1.01	1.29	0.03
Less urban/rural	1.06	0.95	1.18	0.32

OR: Odds Ratio; CI: Confidence Interval.

**Table 4:** Survival.

	All-cause mortality				Cancer mortality				Other-cause mortality			
	HR	95% CI		*P* value	HR	95% CI		*P* value	HR	95% CI		*P* value
		Lower	Upper			Lower	Upper			Lower	Upper	
Age at cancer diagnosis (years)												
66–70	Reference category											
71–75	1.34	1.25	1.44	<0.0001	1.27	1.15	1.40	<0.0001	1.55	1.36	1.75	<0.0001
76–80	1.80	1.68	1.93	<0.0001	1.47	1.34	1.62	<0.0001	2.39	2.12	2.69	<0.0001
>80	3.41	3.20	3.63	<0.0001	2.08	1.90	2.28	<0.0001	5.74	5.15	6.41	<0.0001
Race/ethnicity												
White	Reference category											
Black	1.04	0.97	1.11	0.26	0.91	0.81	1.02	0.09	0.89	0.79	0.99	0.03
Hispanic	0.82	0.74	0.91	<0.01	0.96	0.83	1.12	0.62	0.89	0.76	1.03	0.12
Other	0.79	0.71	0.88	<0.0001	0.94	0.79	1.11	0.45	0.74	0.63	0.89	<0.01
Year of cancer diagnosis												
2001	Reference category											
2002	0.99	0.94	1.04	0.63	0.98	0.90	1.07	0.67	0.99	0.92	1.06	0.67
2003	0.97	0.92	1.02	0.26	1.00	0.91	1.09	0.91	0.88	0.82	0.96	<0.01
2004	0.91	0.86	0.97	<0.01	0.90	0.82	0.99	0.03	0.87	0.79	0.95	<0.01
2005	0.93	0.87	1.00	0.04	0.95	0.86	1.06	0.34	0.83	0.75	0.92	<0.01
Index of prior comorbidity score (NCI Comorbidity Index)												
0	Reference category											
1	1.45	1.39	1.52	<0.0001	1.18	1.10	1.27	<0.0001	1.79	1.68	1.91	<0.0001
≥2	2.05	1.95	2.16	<0.0001	1.34	1.22	1.47	<0.0001	2.86	2.66	3.07	<0.0001
Index of undetected comorbidity score												
0	Reference category											
1	1.45	1.38	1.53	<0.0001	1.32	1.21	1.43	<0.0001	1.58	1.46	1.71	<0.0001
≥2	2.38	2.18	2.60	<0.0001	1.67	1.43	1.95	<0.0001	3.31	2.94	3.73	<0.0001
Indicators of poor performance												
0	Reference category											
≥1	1.80	1.72	1.88	<0.0001	1.41	1.31	1.53	<0.0001	2.15	2.02	2.29	<0.0001
Stage at diagnosis												
*In* *situ*	Reference category											
I	1.79	1.66	1.94	<0.0001	2.66	2.21	3.20	<0.0001	1.44	1.31	1.60	<0.0001
II	2.95	2.73	3.19	<0.0001	7.88	6.59	9.41	<0.0001	1.81	1.63	2.01	<0.0001
III	6.03	5.52	6.58	<0.0001	22.08	18.37	26.54	<0.0001	2.52	2.21	2.86	<0.0001
IV	16.72	15.33	18.23	<0.0001	85.67	71.62	102.47	<0.0001	2.62	2.21	3.10	<0.0001
Histological grade												
Well differentiated	Reference category											
Moderately differentiated	1.11	1.05	1.18	<0.01	1.37	1.22	1.54	<0.0001	1.06	0.98	1.14	0.13
Poorly differentiated	1.47	1.39	1.56	<0.0001	2.28	2.02	2.56	<0.0001	1.14	1.05	1.24	<0.01
Undifferentiated/anaplastic	1.38	1.23	1.56	<0.0001	2.24	1.83	2.74	<0.0001	0.92	0.76	1.11	0.38
Missing/unknown	1.30	1.22	1.40	<0.0001	1.82	1.60	2.07	<0.0001	1.13	1.02	1.24	0.02
Estrogen (ER) and progesterone (PR) receptor status												
ER and PR negative	Reference category											
Either ER or PR positive	0.69	0.65	0.74	<0.0001	0.60	0.54	0.65	<0.0001	0.91	0.81	1.01	0.07
Both ER and PR positive	0.64	0.60	0.67	<0.0001	0.44	0.40	0.47	<0.0001	0.96	0.87	1.05	0.36
Unknown/missing	0.87	0.82	0.93	<0.0001	0.72	0.66	0.79	<0.0001	1.13	1.02	1.26	0.02
Percent in census tract with some college												
<25%	Reference category											
≥25%	1.03	0.99	1.07	0.15	1.05	0.99	1.12	0.09	1.08	1.02	1.15	0.01
Percent in census tract living in poverty												
<5%	Reference category											
5–7%	1.10	1.03	1.16	<0.01	1.11	1.01	1.21	0.03	1.08	0.99	1.17	0.08
8–12%	1.06	1.01	1.12	0.03	0.98	0.90	1.06	0.59	1.03	0.96	1.11	0.40
>12%	1.18	1.12	1.24	<0.0001	1.05	0.97	1.14	0.25	1.06	0.99	1.14	0.10
Type of geographic area												
Large metropolitan	Reference category											
Metropolitan	1.07	1.03	1.12	<0.01	0.99	0.92	1.06	0.70	0.99	0.93	1.05	0.71
Urban	0.97	0.89	1.05	0.38	0.81	0.70	0.93	<0.01	0.76	0.67	0.86	<0.0001
Less urban/rural	1.07	1.00	1.14	0.05	1.03	0.93	1.15	0.56	1.03	0.94	1.14	0.55

HR: Hazard Ratio; CI: Confidence Interval.
